# Reactive Axillary Lymphadenopathy Among Different COVID-19 Vaccines: A Retrospective Study in Breast Sonography

**DOI:** 10.1155/ijbc/8126974

**Published:** 2025-02-15

**Authors:** Pin-Chi Huang, Chia-Hui Chen, Chiao-Hsuan Chien, Chen-Hui Chen, Chin-Yu Chen

**Affiliations:** Department of Radiology, Chi Mei Medical Center, Tainan, Taiwan

## Abstract

**Background/Purpose:** During the coronavirus disease 2019 (COVID-19) outbreak, reactive lymphadenopathy after vaccination is a major concern in breast sonography, especially for patients with a history of breast cancer. The state-of-the-art literature on clinical and sonographic findings either examines a small volume of cases or limited types of severe acute respiratory syndrome coronavirus 2 (SARS-CoV-2) vaccine. This study is aimed at providing vast clinical information to facilitate breast sonographic examination for participants who underwent recent SARS-CoV-2 vaccination.

**Methods:** Among different SARS-CoV-2 vaccines in the Asian Taiwanese population, reactive axillary lymphadenopathy was investigated through breast sonographic findings and clinical data analysis. The sample included participants with recent vaccination by different brands approved in Taiwan, such as the AstraZeneca ChAdOx1 (AZ) vaccine, Moderna mRNA-1273 (Moderna) vaccine, and Pfizer-BioNTech BNT162b2 (BNT) vaccine.

**Results:** A total of 291 participants received the AZ vaccine, 154 received the BNT vaccine, 222 received the Moderna vaccine, and 422 were nonvaccinated during the study period. The incidence rate for axillary reactive lymphadenopathy was 10.9, 21.3, 21.4, and 0.6, respectively. No incidence of malignancy was reported during the 6-month follow-up period. The AZ vaccine, which is a virus-vector vaccine reported a lower incidence rate than mRNA vaccines. We also found lymphadenopathy may last for more than 1 month after vaccination in this study.

**Conclusion:** The study results provide additional supporting information for the management suggested by the recently updated revision of the Society of Breast Imaging guideline pertaining to lymphadenopathy diagnosis of SARS-CoV-2 vaccine–related ipsilateral lymphadenopathy and screening of mammograms.

## 1. Introduction

The coronavirus disease 2019 (COVID-19) outbreak significantly affected the healthcare system and clinical practice, and the country employed massive vaccination to address the COVID-19 pandemic. Unilateral axillary lymphadenopathy is one of the most remarkable side effects of severe acute respiratory syndrome coronavirus 2 (SARS-CoV-2) vaccination [1–3]. By April 17, 2022, a total of 51,063,934 doses of various types of SARS-CoV-2 vaccines were administered to the Taiwanese people. The three kinds of vaccine approved and distributed by the Taiwan Food and Drug Administration (TFDA) are as follows: AstraZeneca ChAdOx1 (AZ) vaccine (15,241,507 doses), Moderna mRNA-1273 (Moderna) vaccine (17,846,369 doses), and Pfizer-BioNTech BNT162b2 (BNT) vaccine (15,671,653 doses) among the 23 million population.

Unfortunately, the SARS-CoV-2 vaccines have a higher incidence of axillary lymphadenopathy compared to other commonly used vaccines such as influenza, human papillomavirus, smallpox, Bacillus Calmette–Guerin, and measles vaccine [4–8]. The presence of unilateral axillary lymphadenopathy caused by SARS-CoV-2 vaccination increased after the mass vaccination [9]. Reactive lymphadenopathy caused by SARS-CoV-2 vaccination is frequently observed in breast imaging studies in axillary regions [10, 11]. However, for patients with a history of breast cancer, an isolated unilateral axillary lymphadenopathy can also be an alarming sign during breast cancer follow-up [1, 12]. Breast sonography is adjunctly used to mammographic screening in dense breasts and is an important diagnostic tool for breast cancer screen and posttreatment follow-up [13]. In most cases, unilateral enlarged axillary lymph nodes may lead to diagnostic dilemmas and result in screening callbacks, additional workups, and invasive tissue diagnosis with fine-needle aspirations or core-needle biopsies [14]. Thus, thorough knowledge and experience in axillary sonography findings after SARS-CoV-2 vaccination are essential to prevent misdiagnosis and further tedious, invasive procedures for nonmetastatic reactive lymph nodes [1, 15]. Since SARS-CoV-2 gradually evolved and may continue to exist worldwide [16], SARS-CoV-2 vaccines will still be needed in the future. Therefore, understanding the influence and mechanisms of SARS-CoV-2 vaccine–related axillary lymphadenopathy is important among breast cancer patients.

## 2. Materials and Methods

### 2.1. Study Design and Sample

The study included female patients who underwent breast sonography in the radiology department of Chi Mei Medical Center (Tainan, Taiwan) between July 2021 and March 2022. The patients' breast cancer history and COVID-19 vaccination history were collected and analyzed. Of the 1212 sequential exams of breast sonography, a total of 740 patient data with a recent history of SARS-CoV-2 vaccination (i.e., within 90 days before the breast sonography examination) were collected.

The following participants were excluded from our study: (1) patients who had COVID-19 vaccination other than the four vaccines approved by TFDA (namely, AZ, BNT, and Moderna), (2) patients who are undergoing neoadjuvant or adjuvant therapy for primary breast cancer with no definitive surgery yet, and (3) patients with breast malignancy other than primary breast cancer.

**Figure 1:**
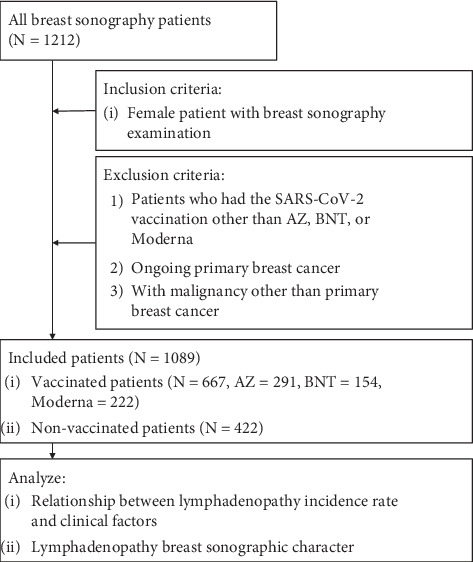
Flow diagram showing the inclusion and exclusion criteria and the vaccine grouping. Abbreviations: *N*: number, AZ: AstraZeneca ChAdOx1 vaccine, BNT: Pfizer-BioNTech BNT162b2 vaccine, Moderna: Moderna mRNA-1273 vaccine, TFDA: Taiwan Food and Drug Administration.

A total of 694 vaccinated patients were included in our study. Of these, 135 patients who had axillary lymphadenopathy after vaccination were divided into four groups based on the type of vaccine, namely, “AZ,” “BNT,” and “Moderna.” A detailed view of the flowchart is shown in Figure 1.

### 2.2. Analysis of Breast Sonography

The sonography results were carefully evaluated by five radiologists specialized in reading breast sonograms with more than 10 years of experience in breast subspecialty. The sonograms were obtained by one of the following high-end ultrasound machines, namely, Aixplorer (SuperSonic Imagine, Aix-en-Provence, France), Aplio 500 (Canon Medical Systems Corporation, Tokyo, Japan), and ACUSON Sequoia Ultrasound System (Siemens Medical Solutions, United States) with a 14–18-MHz linear transducer.

Lymphadenopathy is defined as an enlarged lymph node with cortical thickening > 3 mm, either concentric or eccentric, with or without effacement of fatty hilum, or increased nonhilar vascularity on color or power Doppler [17]. The positive axillary lymph node findings were correlated by confirming a link to SARS-CoV-2 vaccination history within 90 days in the ipsilateral arm.

### 2.3. SARS-CoV-2 Vaccination

Vaccination was usually administered in the deltoid muscle of the nondominant arm. If patients had a history of mastectomy, the vaccinations were administered in the deltoid muscle contralateral to the operated side. Breast sonography exam referrals were based on various breast-related indications other than vaccination-induced complications.

### 2.4. Statistical Analysis

All statistical analyses were performed using the R Stats package [18]. Descriptive summaries were reported as mean ± standard deviation for continuous variables and percentages for categorical variables. For nonparametric variables, median with interquartile range and Mann–Whitney *U* test were used. Statistical significance was set a *p* value < 0.05. Survival analysis for lymphadenopathy-free patients was calculated and graphed using dplyr, survival, and survminer packages in R. The incidence rate was presented as 100-person/month, and the month was defined as 30 days. The incidence rate was calculated using rate ratio test package in R.

## 3. Results and Discussion

### 3.1. Demographic Data

A total of 1089 patients were included in the study. Among them, 667 patients (62.2%) received SARS-CoV-2 vaccination within 90 days prior to the sonography examination. Of these, 297 (42.8%) had a history of breast cancer, and 138 (19.9%) had unilateral axillary lymphadenopathy. Among patients with lymphadenopathy, 35 patients (25.4%) were symptomatic (focal painful swelling) but the result showed no significant difference between groups. No patient in the nonvaccinated group presented symptomatic lymphadenopathy (Table 1). No patient presented fever after vaccination. In the nonvaccinated group, 173 patients (41%) had a history of breast cancer, while nine patients (2.1%) had unilateral axillary lymphadenopathy.

### 3.2. Lymphadenopathy Incidence Rate Among Groups

Among the vaccinated groups, the Moderna group had significantly older participants, and more patients with a history of breast cancer were observed. The nonvaccinated group had a significantly longer follow-up duration. The incidence rate of lymphadenopathy for AZ, BNT, Moderna, and nonvaccinated groups is 10.9, 21.3, 21.4, and 0.6 per 100-person/month, respectively. Additional details are presented in Table 2.

**Figure 2:**
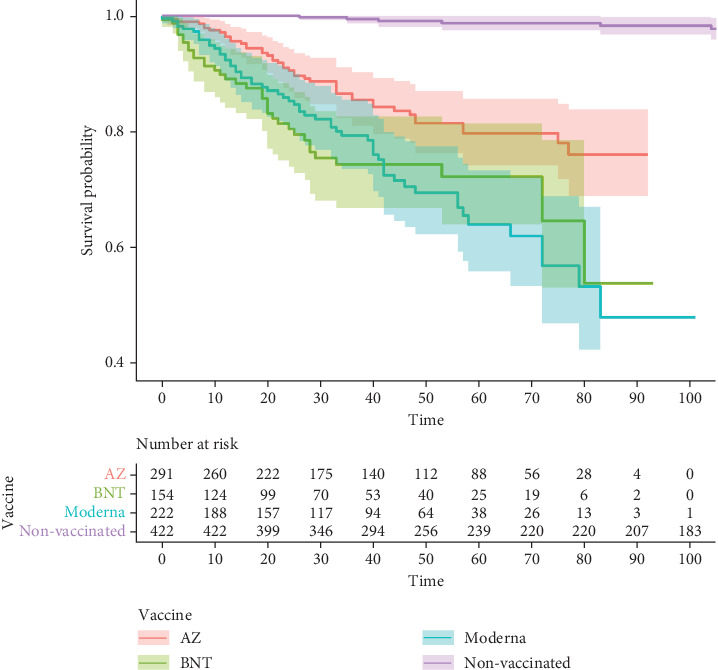
Kaplan–Meier Estimate for lymphadenopathy. Abbreviations: AZ: AstraZeneca ChAdOx1 vaccine, BNT: Pfizer-BioNTech BNT162b2 vaccine, Moderna: Moderna mRNA-1273 vaccine.

**Figure 3:**
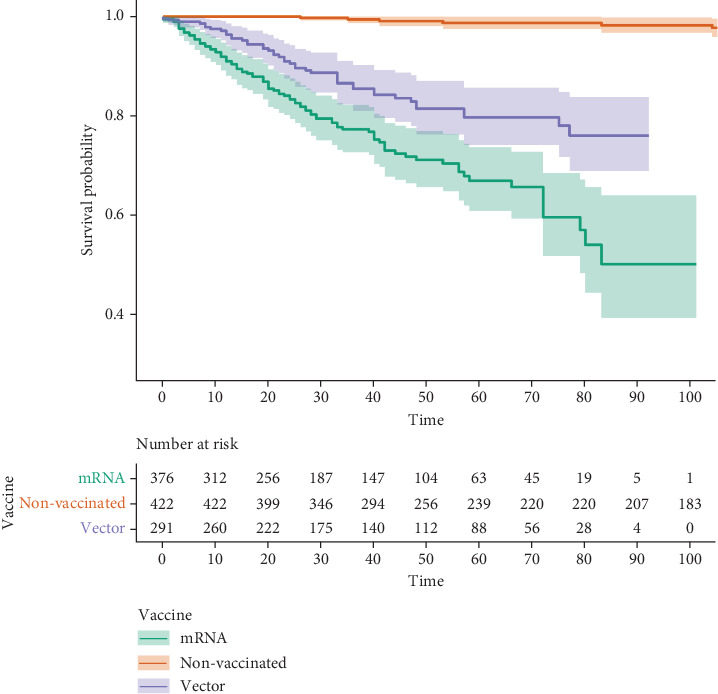
Kaplan–Meier estimate of lymphadenopathy according to immunization mechanism. Abbreviations: AZ: AstraZeneca ChAdOx1 vaccine, BNT: Pfizer-BioNTech BNT162b2 vaccine, Moderna: Moderna mRNA-1273 vaccine. mRNA group equals BNT and Moderna groups. Vector group equals AZ group.

The nonvaccinated groups had significantly lower lymphadenopathy incidence rates when compared with all the other vaccinated groups (*p* < 0.001). The AZ group had a significantly lower incidence rate when compared with BNT (*p* = 0.005) and Moderna vaccines (*p* < 0.001). The Kaplan–Meier curves are illustrated in Figures 2 and 3. Moreover, no patient with lymphadenopathy was clinically proven to have a new onset or recurrent breast cancer diagnosed during the 6-month follow-up period. The follow-up for clinical symptoms and biopsy for persistent lymphadenopathy were conducted in seven patients and revealed a negative finding for malignancy.

As for underlying systemic disease, six patients (2.1%) had a history of autoimmune diseases, all of whom received the AZ vaccine by national health department recommendations at that time. This group included three patients with systemic lupus erythematosus (SLE), two with sicca syndrome, and one with rheumatoid arthritis. Importantly, none of these patients exhibited signs of lymphadenopathy before vaccination. After vaccination, only one patient with SLE, who had stable disease control beforehand, developed lymphadenopathy. This condition appeared 5 days after vaccination and lasted for 36 days before resolving spontaneously. The patient with ongoing breast malignancy and a history of other malignancy is excluded in this study, and we did not find any patient with other systemic disease at risk of lymphadenopathy included.

### 3.3. Sonographic Result

**Figure 4:**
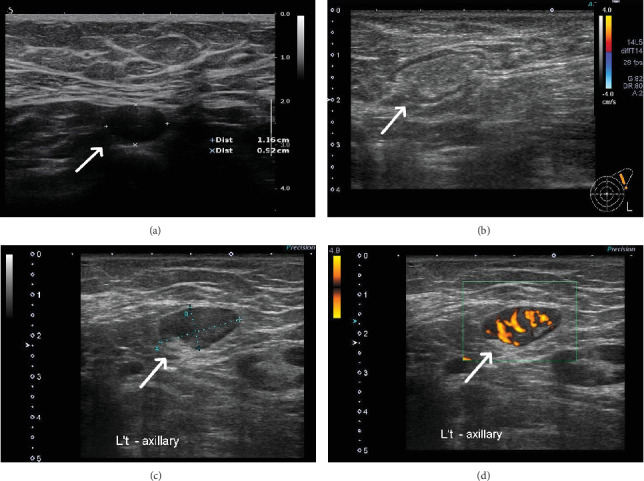
Example for vaccine-related lymphadenopathy. (a) A 50-year-old woman with vaccine-related lymphadenopathy presented as left axillary Level I lymph node with thickened cortex and loss of the normal fatty hilum. (b) Normal lymph node. (c) A 42-year-old woman with vaccine-related lymphadenopathy presented as left axillary Level I lymph node with thickened cortex. (d) Colored Doppler, hypervascularity was presented.

Lymphadenopathy typically lasts for a median of 21 days after vaccination (Q1–Q3: 11–36 days) (Table 1). Based on sonographic data, fewer patients in the AZ group had normal fatty hilum loss. There was no significant difference in the short-axis diameter, cortical thickness, hypervascularity, axillary level, and Breast Imaging Reporting and Data System category among the various groups. A summary of these parameter values is presented in Table 1 and Figure 4.

When comparing vaccination and breast cancer history, the Moderna group is older, and the AZ group was found to be more delayed in lymphadenopathy than the nonvaccinated group (*p* = 0.008). The incidence rate of lymphadenopathy is significantly lower when compared with the BNT and Moderna groups with or without a history of breast cancer (*p* values with breast cancer = 0.095 and 0.044; *p* values without breast cancer = 0.029 and 0.025). However, no significant difference was observed between the BNT and Moderna groups (*p* value with breast cancer = 0.95 and *p* value without breast cancer = 1). For all patients with and without breast cancer, the incidence rate of lymphadenopathy is 18.5 and 14.8 per 100-person/month, respectively (*p* value = 0.228). More detailed information is provided in Table 3.

Many patients received AZ and Moderna as the first dose of SARS-CoV-2 vaccine, while nobody received AZ as the third dose of vaccine during the inclusion period. The disproportional result is related to availability of the vaccines during early days of the pandemic. The incidence of lymphadenopathy was lower when the first dose was the AZ vaccine compared to the BNT (*p* = 0.03) and Moderna (*p* = 0.02) vaccines. Regarding the timing of lymphadenopathy, when the BNT vaccine was administered as the second dose, the onset was more delayed (*p* = 0.028). Fewer patients with lymphadenopathy (*p* = 0.024) were recorded for the third dose of the Moderna vaccine. For groups without a breast cancer history, in the third dose in the Moderna group, the participants were younger (*p* < 0.001). The second dose in the BNT group had a lesser incidence of lymphadenopathy (*p* = 0.04), while the onset was earlier in the Moderna group in the third dose (*p* = 0.013). A summary of these values for the different vaccine groups is presented in Table 4.

When lymphadenopathy is compared based on mechanism of immunization, the vector group (AZ vaccine) showed a significantly lower incidence rate compared to the mRNA group (BNT and Moderna vaccines). Although the results had a biased background clinical condition considering various factors, such as the history of breast cancer, vaccine dosage, and follow-up duration due to the national vaccination policy and vaccine availability, the data still reflect the nature of mechanism of immunization in the real-world setting. A large study comparing the reaction of the different mechanisms of immunization is necessary for future studies.

## 4. Discussion

This study demonstrates the incidence of lymphadenopathy in a real-world clinical setup in Taiwan between July 2021 and March 2022.

The basic demographic data between the different vaccine groups are not evenly distributed in the study but no significant difference in baseline conditions noted between vaccinated groups and nonvaccinated group. This reflects the real-time, real-world medical scenario, given the country's vaccine availability during the first 2 years of the COVID-19 outbreak in Taiwan. The incidence rates of unilateral axillary lymphadenopathy were always significantly higher than the nonvaccinated group in irrespective types of SARS-CoV-2 vaccine administered, as shown in Table 2.

Lymphadenopathy is a sign observed in infection, inflammation, and malignancy. Thus, vaccine-related axillary lymphadenopathy may lead to misdiagnosis and is especially challenging in the case of breast cancer patients with a regular sonographic follow-up. Misinterpretation may occasionally lead to additional imaging studies and unnecessary invasive examinations [19]. Furthermore, in patients with a history of breast cancer and on remission, the incidence rate of lymphadenopathy was not affected. Eventually, none of the 138 patients with a new diagnosis of lymphadenopathy found during the study period was later proven to have a newly diagnosed breast cancer. Similar result was also found by Hagen et al., and none of the included patient was eventually diagnosed with a malignancy after fine-needle aspiration [20]. This finding may provide some support for the recently revised Society of Breast Imaging (SBI) guideline's recommendation on unilateral SARS-CoV-2 vaccine–related lymphadenopathy [21].

With respect to the onset duration of SARS-CoV-2-vaccine-related lymphadenopathy, this study showed that lymphadenopathy may be found at a median 34.5 days after vaccination. Lane et al. found that the BNT and Moderna vaccine–related lymphadenopathy may last for a mean of 117 and 137 days, respectively, before resolution [22]. Some patients may even have axillary lymphadenopathy up to 43 weeks [23]. As lymphadenopathy may be presented weeks late after vaccination, delaying mammograms due to SARS-CoV-2 vaccine–related lymphadenopathy is not clinically appropriate for patients suspicious of new-onset or recurrent breast cancer. These data may be supporting information for the recent SBI guideline recommendation for not postponing sonographic examination due to SARS-CoV-2 vaccination [21].

The incidence rate for each group is as follows: 0.6, 10.9, 21.3, and 21.4 100-person–month incidence rate for the nonvaccinated group, AZ, BNT, and Moderna vaccines, respectively, as presented in Table 2. The incidence rates for BNT and Moderna groups were significantly higher than those for the AZ and nonvaccinated groups. Likewise, the incidence in the nonvaccinated group is significantly lower than that in any vaccine group. The results suggest that all SARS-CoV-2 vaccines increase the risk of vaccine-related lymphadenopathy. Recent 18F-fluorodeoxyglucose PET/CT studies also showed both virus-vector and mRNA vaccines are associated with hypermetabolic lymphadenopathy [24, 25].

In our study, immunization by the vector vaccine group (namely, AZ vaccine) had a significantly lower incidence rate of lymphadenopathy compared to the mRNA vaccine group (i.e., BNT and Moderna vaccines). From previous studies, it is known that the AZ vaccine uses ChAdOx1 viral vector with spike protein DNA to induce an immune response [26], while the BNT and Moderna vaccines use virus mRNA [27, 28]. The difference in immune response may be explained by the difference in incidence rates. Ha et. al found different results between the vector vaccine and mRNA vaccines in the sonographic feature and temporal change of lymph node count [29], Chiang et. al found mRNA vaccines had a higher glucose hypermetabolism than vector vaccine [25], and Jim et. al found mRNA vaccines were associated with a higher COVID-19 vaccine–related lymphadenopathy rate [24].

With respect to sonographic features, the loss of fatty hilum feature presented less in the AZ group. All vaccine groups reported a mild increase in the lymph node axis and a high prevalence of change in cortical thickness and hypervascularity between groups. However, no significant difference was found between the groups. The findings suggest that the loss of fatty hilum may be a specific characteristic for the AZ group. Several previous studies also reported fatty hilum loss, round shape, and change in cortical thickness after different kinds of SARS-COV-2 vaccines [30–32]. Nevertheless, no direct comparison exists in breast sonograms between the different SARS-COV-2-vaccinated patients available in the literature.

## 5. Limitations of the Research Work

This is a retrospective study with a very limited number of SARS-CoV-2 vaccine–related lymphadenopathy patients in a single institution. The single institutional design and the very limited inclusion number are at risk of selection bias, and the result needs to be interpreted carefully. Moreover, during the inclusion period, only a small percentage of the study population received the third dose of the SARS-CoV-2 vaccine as the administration of the third dose was just initiated.

## 6. Conclusions

This study may support management suggestions in recent revision of the SBI guideline recommendations regarding the diagnosis of SARS-CoV-2 vaccine–related ipsilateral lymphadenopathy and screening of mammograms for breast cancer. On comparison of various vaccine groups, all vaccine groups may increase the risk of lymphadenopathy and the virus-vector vaccine had a lower lymphadenopathy incidence rate than mRNA vaccines in this single institute study. These findings suggest future studies with larger study population to aid the discovery of the underlying mechanisms in vaccine-related reactive lymphadenopathy are worthwhile in the future.

## Figures and Tables

**Table 1:** Clinical profile for patients with lymphadenopathy.

		**AZ**		**BNT**		**Moderna**		**p** ** value**
*N*		44		36		58		
Female sex (*N*, %)		44	(100)	36	(100)	58	(100)	
Age (median, Q1–Q3)		51	(44–57)	51	(38–58)	61	(54–66)	< 0.001
Dose (*N*, %)	1st	26	(59.1)	15	(41.7)	34	(58.6)	0.0329
	2nd	18	(40.9)	13	(36.1)	15	(25.9)	
	3rd	0	(0)	8	(22.2)	9	(15.5)	
LAP found after vaccination (days, median, Q1–Q3)		22	(15–33)	17	(8–27)	22	(12–40)	0.968
LAP duration (days, median, Q1–Q3)		23	(13–36)	13	(5.75–25.25)	36	(24.5–41.75)	0.136
Symptomatic (*N*, %)		9	(20.5)	6	(16.7)	20	(34.5)	0.103
Breast malignancy posttreatment history (*N*, %)		17	(31.8)	13	(36.1)	34	(58.6)	0.018
Autoimmune disease history (*N*, %)		1	(2.3)	0		0		
Short-axis diameter (cm, median, Q1–Q3)		0.75	(0.7–0.9)	0.8	(0.7–0.9)	0.8	(0.7–0.9)	0.931
Thickened cortex (*N*, %)		39	(88.6)	32	(88.9)	58	(100)	0.902
Loss of the normal fatty hilum (*N*, %)		2	(4.5)	11	(30.6)	12	(20.7)	0.007
Hypervascularity (*N*, %)		16	(36.3)	13	(36.1)	27	(46.6)	0.837
Axillary level (*N*, %)	I	30	(68.2)	21	(58.3)	41	(70.7)	0.386
	I + II	14	(31.8)	14	(38.9)	17	(29.3)	
	I + II + III	0	(0)	1	(2.8)	0	(0)	
LN_BiRADS category (*N*, %)	2	12	(27.3)	10	(27.8)	9	(15.5)	0.537
	3	32	(72.7)	25	(69.4)	46	(79.3)	
	4a	0	(0)	1	(2.8)	3	(5.2)	

Abbreviations: %, percentage; 1st, first; 2nd, second; 3rd, third; AZ, AstraZeneca ChAdOx1 vaccine; BiRADS: Breast Imaging Reporting and Data System; BNT, Pfizer-BioNTech BNT162b2 vaccine; LN, lymph node; mo, month; Moderna, Moderna mRNA-1273 vaccine; *N*, number; Q1, first quantile; Q3, third quantile.

**Table 2:** Demographic data for all included patients.

		**AZ**		**BNT**		**Moderna**		**Nonvaccinated**	
*N*		291		154		222		422	
Sex (*N*, %)		291	(100)	154	(100)	222	(100)	422	(100)
Age (median, Q1–Q3)		49	(44–56)	47	(39.25–56)	63	(54–68)	51.5	(42–61)
Breast cancer history (*N*, %)		110	(37.8)	49	(31.8)	125	(56.3)	173	(41)
Autoimmune disease history (*N*, %)		6	(2.1)	0		0		0	
Vaccine dose (*N*, %)	1st	144	(49.5)	51	(33.1)	100	(45)		
	2nd	147	(50.5)	69	(44.8)	74	(33.3)		
	3rd	0	(0)	34	(22.1)	48	(21.7)		
Follow-up (days, median, Q1–Q3)		37	(21–64.5)	27.5	(13–50.75)	33	(17–54)	83	(35–174)
LAP IR (*N*, 100-person/mo)		44	(10.9)	36	(21.3)	58	(21.4)	9	(0.6)

Abbreviations: %, percentage; AZ, AstraZeneca ChAdOx1 vaccine; BNT, Pfizer-BioNTech BNT162b2 vaccine; IR, incidence rate; LAP, lymphadenopathy; mo, month; Moderna, Moderna mRNA-1273 vaccine; *N*, number; Q1, first quantile; Q3, third quantile.

**Table 3:** Breast cancer history and SARS-CoV-2 vaccination.

**Breast cancer history**		**AZ**		**BNT**		**Moderna**		**p** ** value**
Positive	Total *N*	110		48		125		
	Age (median, Q1–Q3)	48	(42–54)	44	(37–52)	58	(43–65)	< 0.0001
	Follow-up duration (median, Q1–Q3)	34	(15.5–57)	28	(13.5–53)	36	(17–55)	0.528
	LAP IR (*N*, 100-person/mo)	17	(12.1)	13	(22.1)	34	(22.5)	

Negative	Total *N*	182		106		97		
	Age (median, Q1–Q3)	48	(42–54)	44	(37–52)	58	(43–65)	< 0.0001
	Follow-up duration (median, Q1–Q3)	40	(22–67.25)	28	(12–50)	34.5	(17.75–56)	0.001
	LAP IR (*N*, 100-person/mo)	27	(10.2)	23	(20)	24	(20.1)	

Abbreviations: AZ, AstraZeneca ChAdOx1 vaccine; BNT, Pfizer-BioNTech BNT162b2 vaccine; IR, incidence rate; LAP, lymphadenopathy; mo, month; Moderna, Moderna mRNA-1273 vaccine; *N*, number; Q1, first quantile; Q3, third quantile.

**Table 4:** Lymphadenopathy incidence and SARS-CoV-2 vaccine sequences.

**Vaccine**		**AZ**	**BNT**	**Moderna**	**p** ** value**
1st	Total *N*	144	51	100	
	Breast cancer history (*N*, %)	9 (6.3)	3 (5.9)	22 (22)	< 0.001
	Days after vaccination (median, Q1–Q3)	35.5 (20–63)	25 (14–40.5)	39 (20–55.25)	0.026
	LAP (*N*, %)	26 (18.1)	15 (29.4)	34 (34)	
	IR (100-person/mo)	13.7	29.8	26	

2nd	Total *N*	147	69	74	
	Breast cancer history (*N*, %)	8 (5.4)	9 (13)	10 (13.5)	0.003
	Days after vaccination (median, Q1–Q3)	42 (22.5–67.5)	33 (17–66)	37.5 (19–56)	0.278
	LAP (*N*, %)	18 (12.2)	13 (18.8)	15 (20.2)	
	IR (100-person/mo)	8.3	14.3	15.5	

3rd	Total *N*		34	48	
	Breast cancer history (*N*, %)		1 (2.9)	2 (4.2)	0.362
	Days after vaccination (median, Q1–Q3)		23 (4–43)	25.5 (9–41)	0.603
	LAP (*N*, %)		8 (23.5)	9 (18.8)	
	IR (100-person/mo)		22.3	20.1	

Abbreviations: AZ, AstraZeneca ChAdOx1 vaccine; BNT, Pfizer-BioNTech BNT162b2 vaccine; IR, incidence rate; LAP, lymphadenopathy; mo, month; Moderna, Moderna mRNA-1273 vaccine; *N*, number; Q1, first quantile; Q3, third quantile.

## Data Availability

The clinical information and examination data used to support the findings of this study are available from the corresponding author upon request.
